# Preoperative palsy score has no significant association with survival in non-small-cell lung cancer patients with spinal metastases who undergo spinal surgery

**DOI:** 10.1186/s13018-015-0291-8

**Published:** 2015-09-17

**Authors:** Yen-Jen Chen, Hsien-Te Chen, Horng-Chaung Hsu

**Affiliations:** Department of Orthopedic Surgery, China Medical University Hospital Taichung, Taiwan, No. 2, Yuh-Der Road, Taichung, 404 Taiwan; Department of Orthopedic Surgery, School of Medicine, China Medical University, Taichung, Taiwan; Department of Public Health and Department of Health Services Administration, China Medical University, Taiwan, No. 91, Hsueh-Shuh Road, Taichung, 404 Taiwan

**Keywords:** Lung cancer, Neurological status, Prognostic score, Spinal metastasis, Survival rate

## Abstract

**Background:**

Survival is a key factor physicians consider when selecting a treatment modality for the treatment of spinal metastases. Various assessment systems can predict length of survival and facilitate selection of the most appropriate treatment. Spinal palsy is a prognostic parameter in the Tokuhashi scoring system but not in the Tomita scoring system. A limitation of these scoring systems is that studies of them have included different tumor types. The aim of this study was to evaluate the usefulness of preoperative neurological status as a prognostic factor in non-small-cell lung cancer patients with spinal metastases who underwent surgical treatment.

**Methods:**

From November 2000 to March 2010, 50 patients with symptomatic metastatic spinal cord compression secondary to non-small-cell lung cancer underwent palliative surgery. Data collected included patient age and sex, tumor histology, date of surgery, death or last follow-up, preoperative and postoperative ambulatory status according to the Frankel grading system, body mass index (BMI), number of vertebra involved, number of other bone metastasis, visceral metastasis, and preoperative Karnofsky performance status. Log-rank test and multivariate Cox proportional hazard regressions were used to evaluate possible prognostic factors.

**Results:**

The mean patient age was 61.6 years (range, 20–87 years), and 34 were male and 16 were female. The median postoperative survival time was 7.5 months. The median survival was 2.5 months (95 % confidence interval (CI): 1.22–16.3 months) in the Frankel A + B group and 8.0 months (95 % CI: 5.52–9.89 months) in the Frankel C + D group (*p =* 0.87). Multivariate Cox proportional hazard regressions showed that preoperative performance status was significantly associated with survival. Preoperative palsy score had no statistically significant association with survival.

**Conclusions:**

Preoperative palsy score had no statistically significant association with survival in non-small-cell lung cancer patients with spinal metastases who underwent spinal surgery in this study.

## Background

As advances in chemotherapy prolong the life expectancy of patients with solid tumors, the frequency of spinal metastases is likely to increase. Survival is a key factor physicians consider when selecting a treatment for spinal metastases. Various assessment systems can predict the length of survival and facilitate the selection of the most appropriate treatment. The assessment systems, however, differ with respect to the parameters assessed and the significance assigned to each parameter in the total score. Spinal palsy is one of the prognostic parameters in the Tokuhashi scoring system [1, 2] but is not included in the Tomita scoring system [3]. A limitation of these scoring systems is that studies examining them have included different tumor types.

The aim of this study was to evaluate the usefulness of preoperative neurological status as a prognostic factor in non-small-cell lung cancer patients with spinal metastases who underwent surgical treatment.

## Material and methods

### Patients

From November 2000 to March 2010, 50 patients with symptomatic metastatic spinal cord compression secondary to non-small-cell lung cancer underwent palliative surgery. A retrospective review of the hospital records and radiographs of these patients was conducted. The indication for surgery was neurologic deficit due to spinal cord compression. A single surgeon performed all the surgeries. The Research Ethics Committee (China Medical University & Hospital, Taichung, Taiwan) approved this retrospective analysis (No. DMR101-IRB2-310).

All patients presented with weakness in the lower extremities, and 10 patients (20 %) remained ambulatory. The Frankel grading system [4] and a supplementary ambulatory status score were used during the preoperative and postoperative periods to evaluate neurologic status. Preoperative evaluations included plain radiographs and magnetic resonance imaging (MRI) or computed tomography (CT).

### Surgical interventions

A total of 55 surgical procedures were performed on the 50 patients, and 46 patients underwent a single operation. Three patients underwent primary surgery for metastatic spinal cord compression, followed by a second operation for noncontiguous metastases. One patient underwent 3 additional procedures because of repeated local recurrences over a 3-year period.

Three patients underwent combined anterior and posterior procedures (anterior corpectomy, reconstruction with polymethylmethacrylate, and posterior instrumentation). Two patients with cervical spine metastases underwent anterior surgery. The remaining 45 (90 %) patients underwent a posterolateral transpedicle approach (PTA). All patients underwent spinal instrumentation following adequate decompression. Local radiotherapy, systemic chemotherapy, and/or targeted therapy were performed after wound healing, usually 3–4 weeks after surgery.

### Factors for analysis

Data collected included patient age and sex, tumor histology, date of surgery, death or last follow-up, preoperative and postoperative ambulatory status according to the Frankel grading system, body mass index (BMI), number of vertebra involved, number of other bone metastasis, visceral metastasis, and preoperative Karnofsky performance status. Overall survival was calculated from the date of surgery to the date of death.

Factors included in the analyses were sex, age (≤54, 55–74, and ≥75 years), tumor type (adenocarcinoma or nonadenocarcinoma), preoperative and postoperative palsy score (Frankel A + B vs. Frankel C + D vs. Frankel E), BMI (underweight vs. eutrophic vs. overweight/obese), number of vertebra involved (<3 vs. ≥3), other bone metastasis (without vs. with), visceral metastasis (without vs. with), and preoperative Karnofsky performance status (10–40 % vs. 50–70 % vs. 80–100 %).

### Statistical analysis

Survival curves were plotted using the Kaplan-Meier method, and the significance of the differences between groups was determined using a log-rank test that considered the effects of age. The median survival time and 95 % confidence interval (CI) were then estimated based on the Brookmeyer and Crowley method [5]. A *p* value <0.05 was considered statistically significant. Chi-squared test statistics and *p* values were calculated based on the log-rank test of specific pairs. For variables with 2 subgroups, a *p* < 0.05 was considered statistically significant. For variables with 3 subgroups, a *p* < 0.0167 was considered statistically significant (the Bonferroni correction method was used to suppress a spurious significant difference).

Univariate and multivariate Cox proportional hazard regressions were used to detect possible prognostic factors. To investigate the most significant factors, factors significantly impacted with survival in univariate analysis were included in multivariate analysis. Pre-op palsy score was considered to be the important factor, so it was included in multivariate analysis even no significance in univariate analysis. A *p* < 0.05 was considered statistically significant. All analyses were performed using SAS 9.1 statistical software (SAS Institute, Inc, Cary, NC, USA).

## Results

The mean patient age was 61.6 years (range, 20–87 years), and there were 34 males and 16 females. The tumor sites included the thoracic spine (*n* = 28), lumbar spine (*n* = 12), thoracolumbar spine (*n* = 6), cervical spine (*n* = 3), and sacrum (n = 1). Adenocarcinoma (32 patients) was the most common histological type, followed by squamous cell carcinoma (9 patients). The mean intraoperative blood loss volume was 975 mL (range, 350–6500 mL), and the mean surgical time was 4.8 h.

Neurologic improvement by ≥1 Frankel grade was noted in 37 of the 50 cases (74 %). Twelve patients showed no improvement, and 1 patient showed deterioration from Frankel grade B to grade A. Overall, 68 % of patients (34/50) were ambulatory after surgery. Twenty-two of 40 (55 %) nonambulatory (Frankel B/C) patients became ambulatory (Frankel D/E).

One patient developed symptomatic tumor recurrence at the level of previous decompression, and 3 patients developed new symptomatic spinal cord compressions because of noncontiguous metastases. These patients underwent repeat decompressive surgeries.

**Fig. 1:**
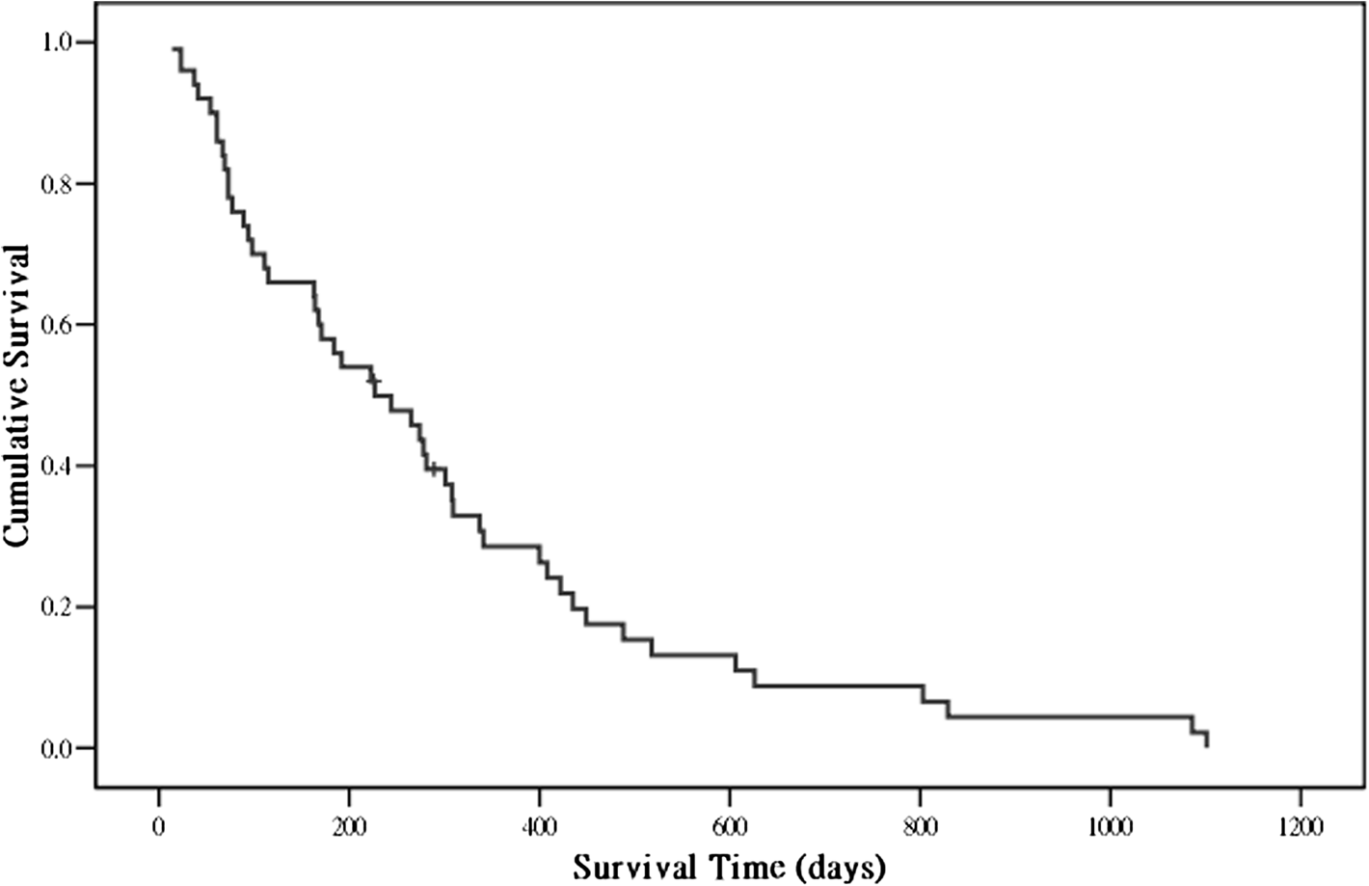
Kaplan-Meier survival curve of the 50 lung cancer patients with spinal metastases who underwent spinal surgery

**Fig. 2:**
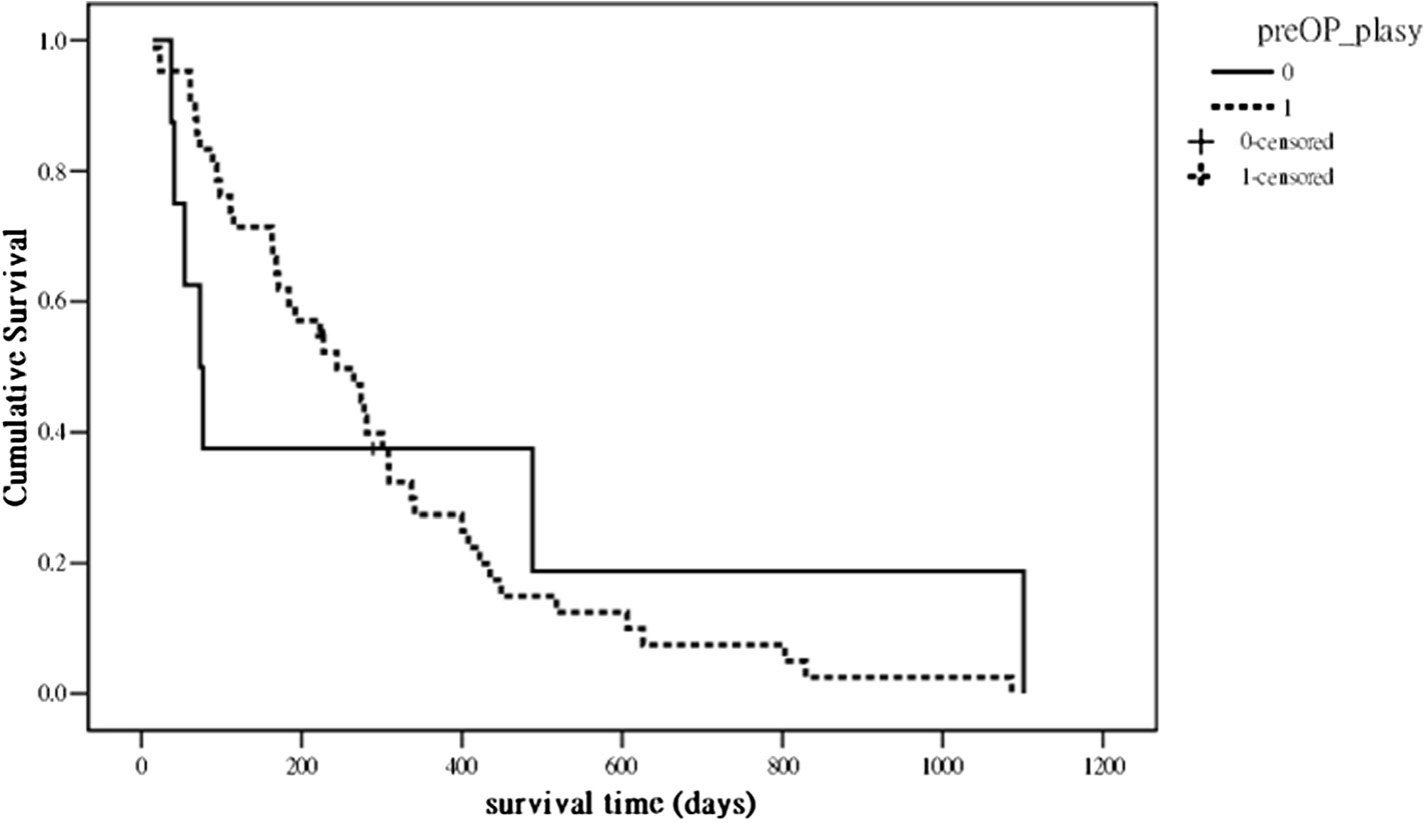
Kaplan-Meier survival curve of the 50 patients with pre-operative Tokuhashi palsy score 0 vs score 1 (*p =* 0.87)

Of 12 observed complications (Table 1), 11 were surgery related. There was no intraoperative mortality; however, 3 patients died during the postoperative period. One patient died from respiratory failure 14 days after surgery, 1 died from hepatic and respiratory failure 23 days after surgery, and 1 died from nonsurgery-related sigmoid colon perforation and sepsis 37 days after surgery. The median postoperative survival time was 7.5 months (95 % CI: 4.2–10.9 months). The Kaplan-Meier curve (Fig. 1) showed that 58 % (29/50) of the patients survived >6 months. The Frankel A + B group (palsy score 0 in Tokuhashi system) contained 8 patients, and the Frankel C + D group (palsy score 1 in Tokuhashi system) contained 42 patients. The median survival was 2.5 months (95 % CI: 1.22–16.3 months) in the Frankel A + B group and 8.0 months (95 % CI: 5.52–9.89 months) in the Frankel C + D group (Fig. 2; *p =* 0.87).

**Table 1:** Post-operative complications

Complication	Number of patients
Neurologic progression	1
Wound dehiscence	1
Wound infection	3
Respiratory failure	2
CSF leakage	2
Sigmoid colon perforation	1
30-day mortality	2

*CSF* cerebrospinal fluid

Log-rank test (Table 2) and pairwise comparisons of survival between subgroups (Table 3) showed that age <75 years, adenocarcinoma histology, higher preoperative performance status score, and higher postoperative palsy score were all significantly associated with longer survival. Sex, BMI, number of vertebra involved, other bone metastasis, visceral metastasis, and preoperative palsy score had no statistically significant association with survival.

**Table 2:** Kaplan-Meier survival curve estimates for analysis of prognostic factors for survival

Variables	Number of patients	Median survival (month)	*p* value^a^
		(95 % CI)	
Age, year			0.010*
≤54	16	9.5 (3.22–11.07)	
55–74	24	8.7 (3.09–13.14)	
≥75	10	3.7 (2.0–5.52)	
Sex			0.220
M	34	6.3 (3.09–9.13)	
F	16	11.1 (5.52–13.86)	
Tumor histology			0.003*
Adenocarcinoma	32	9.9 (7.43–11.20)	
Non-adenocarcinoma	18	3.5 (2.20–5.39)	
Pre-op palsy score			0.870
0 (Frankel 1, 2)	8	2.5 (1.22–16.03)	
1 (Frankel 3, 4)	42	8.0 (5.52–9.89)	
Post-op palsy score			<0.001*
0 (Frankel 1, 2)	2	2.4 (2.4–2.4)^b^	
1 (Frankel 3, 4)	32	5.5 (2.92–7.33)	
2 (Frankel 5)	16	14.3 (9.23–17.02)	
Pre-op PS			<0.001*
Poor (0, PS 10–40 %)	8	2.4 (0.46–3.78)	
Moderate (1, PS 50–70 %)	20	3.7 (2.4–6.3)	
Good (2, PS 80–100 %)	22	13.1 (9.23–16.03)	
BMI			0.540
Underweight (1)	5	6.3 (0.59–12.02)	
Eutrophic (2)	31	6.0 (3.18–8.91)	
Overweight/obese (3)	14	9.1 (7.62–10.65)	
Number of vertebra involved			0.630
<3	24	6.3 (3.15–9.46)	
≥3	26	9.1 (3.16–15.11)	
Other bone metastasis			0.818
Without	20	5.4 (1.25–9.46)	
With	30	8.7 (6.56–10.85)	
Visceral metastasis			0.567
Without	40	8.0 (5.53–10.51)	
With	10	3.2 (0–7.65)	

*Significant at *p* value <0.05

^a^ *p* value is calculated based on log-rank test over all stratification and takes the effect of age into consideration

^b^The 95 % CI may be problematic due to too few data values

*PS* performance status, *CI* confidence interval, *BMI* body mass index

**Table 3:** Pairwise comparisons of survival between subgroups

Variable	Chi-square	*p* value
Histology		
Adeno vs Non-adeno	8.7	0.003*
Sex		
F vs M	1.5	0.220
Age (year)		
≤54 vs 55–74	0.19	0.660
≤54 vs ≥75	7.3	0.007*
55–74 vs ≥75	6.41	0.010*
Pre-op PS		
0 vs 1	0.45	0.500
0 vs 2	13.44	<0.001*
1 vs 2	13.99	<0.001*
Pre-op palsy		
0 vs 1	0.027	0.870
Post-op palsy		
0 vs 1	1.95	0.160
0 vs 2	6	0.010*
1 vs 2	9.12	0.003*
BMI		
1 vs 2	0.02	0.878
1 vs 3	1.07	0.301
2 vs 3	0.97	0.324
Number of vertebra involved		
<3 vs ≥3	0.23	0.630
Other bone metastasis		
Without vs with	0.05	0.818
Visceral metastasis		
Without vs with	0.33	0.567

*Significant at *p-*value <0.05

*Adeno* adenocarcinoma, *Non-adeno* non-adenocarcinoma, *PS* performance status, *BMI* body mass index, *BMI 1* underweight, *2* eutrophic, *3* overweight/obese

Multivariate Cox proportional hazard regressions further showed that only higher preoperative performance status score had a statistically significant association with longer survival (Table 4).

**Table 4:** Univariate and multivariate Cox proportional hazard regressions Model

	Univariate		Multivariate	
Variable	Hazard ratio (95 % CI)	*p* value	Hazard ratio (95 % CI)	*p* value
Sex				
F (ref: M)	0.61 (0.33–1.14)	0.120		
Age (year)				
55–74 (ref: ≤54)	1.16 (0.6–2.25)	0.659	0.78 (0.37–1.64)	0.512
≥75 (ref: ≤54)	3.28 (1.37–7.82)	0.008*	1.22 (0.37–4.05)	0.748
BMI (kg/m^2^)				
Eutrophic (ref: underweight)	1.03 (0.39–2.68)	0.958		
Overweight (ref: underweight)	0.72 (0.25–2.05)	0.538		
Pre-op palsy				
1 (ref: 0)	1.18 (0.49–2.83)	0.706	1.23 (0.5–3.03)	0.653
Post-op palsy				
1 (ref: 0)	0.3 (0.06–1.36)	0.119		
2 (ref: 0)	0.1 (0.02–0.51)	0.006*		
PS score				
1 (ref: 0)	0.43 (0.18–1.03)	0.059	0.52 (0.16–1.74)	0.289
2 (ref: 0)	0.09 (0.03–0.26)	<0.001*	0.14 (0.03–0.54)	0.004*
Histology				
Adeno (ref: Non-adeno)	0.38 (0.2–0.71)	0.003*	0.59 (0.28–1.25)	0.167
Number of vertebra involved				
≥3 (ref: <3)	0.7 (0.39–1.25)	0.228		
Other bone metastasis				
With (ref: without)	0.83 (0.46–1.49)	0.531		
Visceral metastasis				
With (ref: without)	1.08 (0.52–2.23)	0.837		

*Significant at *p-*value <0.05

*PS* performance status, *CI* confidence interval, *ref* reference, *Adeno* adenocarcinoma, *Non-adeno* non-adenocarcinoma, *BMI* body mass index

## Discussion

Tokuhashi et al. [2] stated that the average survival period was longer (10.4 ± 13.6 months) in patients without neurologic deficits than in patients with complete palsy (3.7 ± 3.9 months). Therefore, they included “spinal cord palsy” as a prognostic parameter in their study. Several authors have reported that patients with paralysis at presentation or posttreatment have a considerably shorter life expectancy than ambulatory patients [6–10]. Prasad and Schiff [11] reported that other than the nature of the primary tumor, the presence of paraparesis prior to surgery had the most detrimental effect on survival.

In 2001, Tomita et al. [3] developed a scoring system that does not use neurologic status as a prognostic factor for survival in patients with spinal metastases. The authors described that a long survival period can be possible with appropriate treatment, even in cases with paraplegia, and suggested that spinal cord decompression can improve paralytic conditions. Patients with paralysis tend to have shorter survival because of cancer progression and not due to the paralysis itself [3]. Spiegel et al. [12] reported that neurologic deficits did not significantly influence survival of melanoma patients. North et al. [13] observed that the preoperative ambulatory status predicted the duration of postoperative ambulation but was only marginally associated with survival. Yamashita et al. [14] used the revised Tokuhashi scoring system to predict survival in patients with spinal metastases and found that Frankel grade was not significantly associated with survival. Kumar et al. [15] studied 87 patients with spinal metastases from nasopharyngeal cancer and found the modified Tokuhashi score was the best to predict prognosis; however, neurological status had no significant association with survival. Quraishi et al. [16] studied the effect of surgical timing on neurological outcome and survival in spinal metastases patients and found that earlier surgical treatment resulted in significantly better neurological outcomes. However, the timing of surgery did not influence survival.

Leithner et al. [17] compared 7 preoperative prognostic scoring systems for spinal metastases, including the Bauer [18], modified Bauer [19], Tokuhashi [2], revised Tokuhashi [1], Tomita [3], van der Linden [20], and Sioutos [21] scoring systems. In their analyses, primary tumor and visceral metastases were the only parameters significantly associated with survival. Their results did not show pretreatment neurological status as a prognostic factor; therefore, the authors did not consider paralysis as a predictive of survival [17, 22]. Wibmer et al. [23] evaluated the same preoperative scoring systems and found that primary tumor, status of visceral metastases, and systemic therapy were significantly associated with survival. Leithner et al. [17] and Wibmer et al. [23] further concluded that the Bauer and modified Bauer scoring systems are the most reliable systems for prediction of survival. The modified Bauer scoring system includes 4 positive prognostic factors: absence of visceral metastases, solitary skeletal metastasis, non-primary lung cancer, and primary tumor of the breast or kidney, lymphoma, or myeloma. It does not include preoperative neurological palsy as a parameter.

The parameters of the revised Tokuhashi scoring system include the patient’s general condition, number of extraspinal bone metastases, number of metastases in the vertebral bodies, presence of metastases in the major internal organs, the primary site of the cancer, and the presence of palsy. The first 5 parameters are all associated with disease severity, but palsy score is not. In our study, the survivals of the Frankel A + B and Frankel C + D groups were not statistically different. One patient with preoperative Frankel B status improved to Frankel D postoperatively and survived for 16 months. One patient with preoperative Frankel B status improved to Frankel C postoperatively and survived for 36 months. Patients with preoperative Frankel B neurological status can still survive for a long duration.

**Fig. 3:**
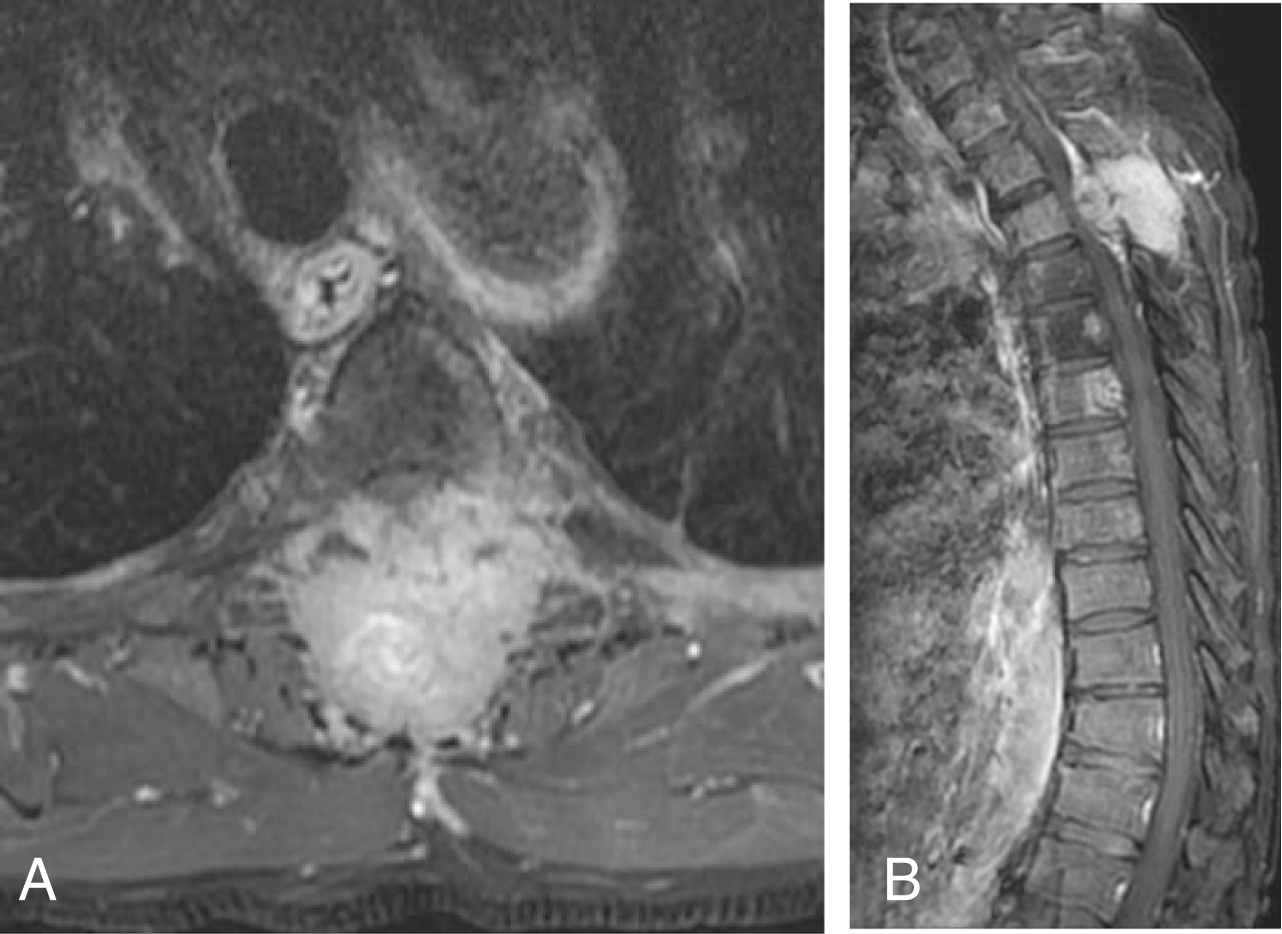
A 69-year-old male lung cancer patient with T3, T6 metastases underwent spinal surgery; his preoperative palsy score was 0 (Frankel B). Axial (**a**) and sagittal (**b**) T1-weighted MR images with contrast enhancement demonstrate severe cord compression at T3. His postoperative palsy score was 1 (Frankel C), and he survived 289 days after spinal surgery

**Fig. 4:**
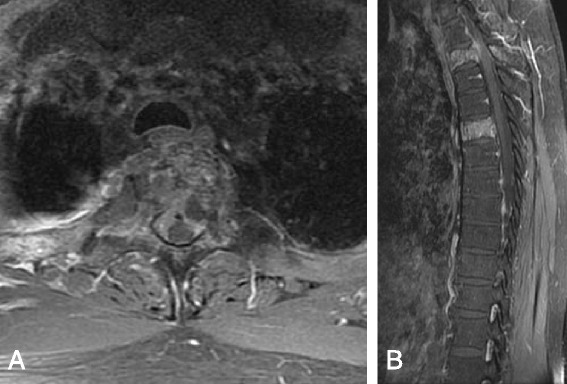
A 51-year-old male lung cancer patient with T2, T5 metastases underwent spinal surgery; his preoperative palsy score was 1 (Frankel D). Axial (**a**) and sagittal (**b**) T1-weighted MR images with contrast enhancement demonstrate cord compression at T2. His postoperative palsy score was 1 (Frankel D), and he survived 274 days after spinal surgery

These results may be because paralytic condition is not associated with disease severity, and paralytic condition can be improved with adequate spinal cord decompression. In a patient with multiple spine metastases, palsy might be absent. However, palsy might be noted in a patient with only 1 vertebral metastasis. A patient with multiple spinal metastases generally has higher disease severity than a patient with a single vertebral metastasis. Also, the number of vertebrae involved and the pattern of spinal cord compression might be the same in patients with different palsy score (Figs. 3 and 4). Deterioration of neurological status might occur in 1 week, and the survival time should not differ too much in such situation. After adequate surgical decompression and stabilization, palsy should be reversed in all patients except those with a neurological status of Frankel A, or those with a poor overall medical status [24]. Thus, palsy should not be a major prognostic factor in lung cancer patients with spinal metastasis who underwent spinal surgery. Patients with paralysis tend to have shorter survival because of cancer progression, not due to the paralysis itself [3]. The duration of survival largely depends on the disease severity and the ability of other modalities (such as chemotherapy, targeted therapy, or radiotherapy) to control the tumor [25–28].

This study has some limitations that should be considered. First, as a retrospective study from a single center, several potential biases may exist, including referral bias and patient characteristics. Second, the sample size was small. Third, we did not include all parameters in the analyses. However, this is the first study that focused only on non-small-cell lung cancer patients with spinal metastases who underwent spinal surgery. The result of this study should be valuable in the decision of treatment of non-small-cell lung cancer patients with spinal metastases.

## Conclusions

Preoperative palsy score had no statistically significant association with survival in non-small-cell lung cancer patients with spinal metastases who underwent spinal surgery.

## Abbreviations

BMI: body mass index
CI: confidence interval
CT: computed tomography
MRI: magnetic resonance imaging
PTA: posterolateral transpedicular approach
